# EEFSEC deficiency: A selenopathy with early-onset neurodegeneration

**DOI:** 10.1016/j.ajhg.2024.12.001

**Published:** 2025-01-02

**Authors:** Lucia Laugwitz, Rebecca Buchert, Patricio Olguín, Mehrdad A. Estiar, Mihaela Atanasova, Wilson Marques Jr., Jörg Enssle, Brian Marsden, Javiera Avilés, Andrés González-Gutiérrez, Noemi Candia, Marietta Fabiano, Susanne Morlot, Susana Peralta, Alisa Groh, Carmen Schillinger, Carolin Kuehn, Linda Sofan, Marc Sturm, Benjamin Bender, Pedro J. Tomaselli, Uta Diebold, Amelie J. Mueller, Stephanie Spranger, Maren Fuchs, Fernando Freua, Uirá Souto Melo, Lauren Mattas, Setareh Ashtiani, Oksana Suchowersky, Samuel Groeschel, Guy A. Rouleau, Keren Yosovich, Marina Michelson, Zvi Leibovitz, Muhammad Bilal, Eyyup Uctepe, Ahmet Yesilyurt, Orhan Ozdogan, Tamer Celik, Ingeborg Krägeloh-Mann, Olaf Riess, Hendrik Rosewich, Muhammad Umair, Dorit Lev, Stephan Zuchner, Ulrich Schweizer, David S. Lynch, Ziv Gan-Or, Tobias B. Haack

**Affiliations:** 1 Institute of Medical Genetics and Applied Genomics, University of Tübingen, 72076 Tübingen, Germany; 2 Neuropediatrics, General Paediatrics, Diabetology, Endocrinology and Social Paediatrics, University of Tübingen, University Hospital Tübingen, 72016 Tübingen, Germany; 3 Department of Neuroscience, Facultad de Medicina, Universidad de Chile, Santiago 8380453, Chile; 4 Program of Human Genetics, Biomedical Sciences Institute, Facultad de Medicina, Universidad de Chile, Santiago 8380453, Chile; 5 Broad Institute of MIT and Harvard, Cambridge, MA, USA; 6 Department of Human Genetics, McGill University, Montreal, QC, Canada; 7 Centre for Medicines Discovery, Nuffield Department of Medicine, University of Oxford, Oxford, UK; 8 Neuroscience and Behavioral Sciences Department, Ribeirão Preto Medical School, University of São Paulo, Ribeirão Preto 14048-900, Brazil; 9 Institut für Biochemie und Molekularbiologie, Uniklinikum Bonn, Rheinische Friedrich-Wilhelms-Universität Bonn, 53115 Bonn, Germany; 10 Department of Human Genetics, Hannover Medical School, Hanover, Germany; 11 Diagnostic and Interventional Neuroradiology, Radiologic Clinics, University of Tübingen, 72076 Tübingen, Germany; 12 Social Pediatric Center, Auf der Bult, Hannover, Germany; 13 MVZ Humangenetik Bremen, Limbach Genetics, 28209 Bremen, Germany; 14 Sozialpädiatrisches Zentrum (SPZ), Allgemeines Krankenhaus Celle, 29221 Celle, Germany; 15 Division of Clinical Neurology, Hospital das Clinicas da Universidade de São Paulo, São Paulo, Brazil; 16 Max Planck Institute for Molecular Genetics, RG Development & Disease, Berlin, Germany; 17 Institute for Medical and Human Genetics, Charité Universitätsmedizin Berlin, Berlin, Germany; 18 Department of Pediatrics, Division of Medical Genetics, Stanford Medicine, Stanford, CA, USA; 19 Alberta Children’s Hospital, Medical Genetics, Calgary, AB, Canada; 20 Departments of Medicine (Neurology) and Medical Genetics, University of Alberta, Edmonton, AB, Canada; 21 The Neuro (Montreal Neurological Institute-Hospital), McGill University, Montréal, QC, Canada; 22 Department of Human Genetics, McGill University, Montréal, QC, Canada; 23 Department of Neurology and Neurosurgery, McGill University, Montréal, QC, Canada; 24 Molecular Genetic Lab, Wolfson Medical Center, Holon 58100, Israel; 25 The Rina Mor Institute of Medical Genetics, Wolfson Medical Center, Holon 58100, Israel; 26 Obstetrics & Gynecology Ultrasound Unit, Bnai Zion Medical Center, Rappaport Faculty of Medicine, Technion-Israel Institute, Haifa, Israel; 27 Department of Pathology and Laboratory Medicine, Aga Khan University, Karachi 74800, Pakistan; 28 Acibadem Labgen Genetic Diagnosis Center, Istanbul, Turkey; 29 Acibadem Maslak Hospital, Istanbul, Turkey; 30 Departman of Pediatric Neurology, Adana City Training and Research Hospital, Adana, Turkey; 31 Center for Rare Disease, University of Tübingen, 72076 Tübingen, Germany; 32 Department of Pediatrics and Adolescent Medicine, Division of Pediatric Neurology, University Medical Center Göttingen, Georg August University, Göttingen, Germany; 33 Medical Genomics Research Department, King Abdullah International Medical Research Center (KAIMRC), King Saud bin Abdulaziz University for Health Sciences, Ministry of National Guard Health Affairs, Riyadh, Saudi Arabia (KSA); 34 Department of Life Sciences, School of Science, University of Management and Technology, Lahore, Pakistan; 35 Institute of Medical Genetics, Wolfson Medical Center, Holon 58100, Israel; 36 The Rina Mor Institute of Medical Genetics, Wolfson Medical Center, Holon 58100, Israel; 37 Dr. John T. Macdonald Foundation Department of Human Genetics, John P. Hussman Institute for Human Genomics, University of Miami Miller School of Medicine, Miami, FL, USA; 38 Department of Neurogenetics, National Hospital for Neurology & Neurosurgery, Queen Square, London, UK; 39 Department of Neuromuscular Diseases, UCL Queen Square Institute of Neurology, London, UK; 40 NIHR University College London Hospitals Biomedical Research Centre, London, UK; 41 Genomics for Health in Africa (GHA), Africa-Europe Cluster of Research Excellence (CoRE)

**Keywords:** cerebellar hypoplasia, progressive spasticity, cerebellar atrophy, epilepsy, EEFSEC deficiency, selenoproteins, selenopathy

## Abstract

Inborn errors of selenoprotein expression arise from deleterious variants in genes encoding selenoproteins or selenoprotein biosynthetic factors, some of which are associated with neurodegenerative disorders. This study shows that bi-allelic selenocysteine tRNA-specific eukaryotic elongation factor (*EEFSEC*) variants cause selenoprotein deficiency, leading to progressive neurodegeneration. EEFSEC deficiency, an autosomal recessive disorder, manifests with global developmental delay, progressive spasticity, ataxia, and seizures. Cerebral MRI primarily demonstrated a cerebellar pathology, including hypoplasia and progressive atrophy. Exome or genome sequencing identified six different bi-allelic *EEFSEC* variants in nine individuals from eight unrelated families. These variants showed reduced EEFSEC function *in vitro*, leading to lower levels of selenoproteins in fibroblasts. In line with the clinical phenotype, an eEFSec-RNAi *Drosophila* model displays progressive impairment of motor function, which is reflected in the synaptic defects in this model organisms. This study identifies EEFSEC deficiency as an inborn error of selenocysteine metabolism. It reveals the pathophysiological mechanisms of neurodegeneration linked to selenoprotein metabolism, suggesting potential targeted therapies.

## Teaser

Bi-allelic variants in *EEFSEC*, essential for selenoprotein biosynthesis, cause a novel selenopathy with early-onset neurodegeneration characterized by developmental delay, spasticity, seizures, and cerebellar atrophy. Functional studies and models reveal impaired selenoprotein translation, synaptic deficits, and progressive motor dysfunction, providing mechanistic insights and therapeutic opportunities for selenium-related neurodegenerative disorders.

## Main text

**Figure 1:**
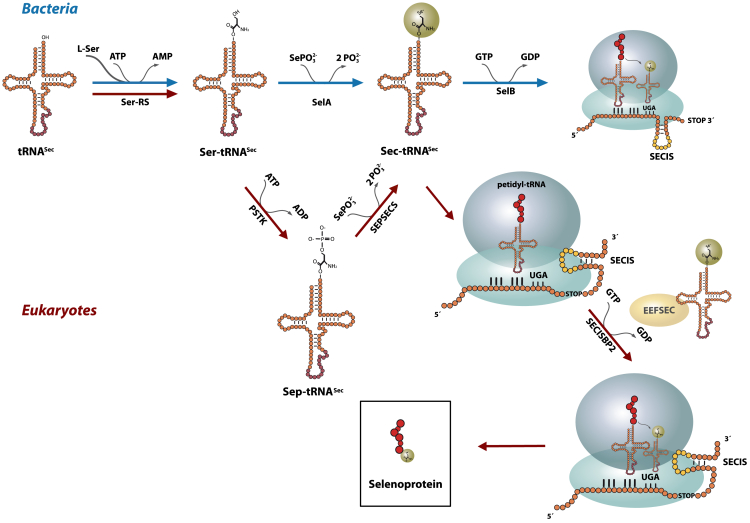
Selenoprotein synthesis in eubacteria and eukaryotes modified according to Simonovic and Puppala In all domains of life, tRNA^Sec^ is serylated by SerRS. In eubacteria (blue), the Ser is converted to Sec by the enzyme SelA. The Sec-tRNA^Sec^ is delivered by SelB^∗^GTP (bacterial analog to EEFSEC) to the bacterial ribosome reading an in-frame UGA. Elongation is favored over termination at the UGA by proteins binding to the selenocysteine insertion sequence (SECIS), a stem-loop structure in selenoprotein mRNAs.^5^^,^^6^^,^^7^^,^^8^^,^^9^^,^^10^ The SECIS element, which is formed by the coding region, prevents binding of a release factor. In eukaryotes (red), the Ser-tRNA^Sec^ is phosphorylated to phosphoserine (Sep) by PSTK. Afterward, SEPSECS converts Sep-tRNA^Sec^ into Sec-tRNA^Sec^ using selenophosphate. EEFSEC^∗^GTP brings Sec-tRNA^Sec^ into the A-site of a ribosome to read the UGA codon. The SECIS element is located in the 3′ UTR of the mRNA and interacts with EEFSEC and SECISBP2 in a complex on the ribosome.^5^ Peptidyl transfer of the peptide forms the P-site tRNA on Sec-tRNA^Sec^ in the A-site, incorporating Sec into the nascent selenoprotein.

The mammalian brain depends on the incorporation of the essential trace element selenium (Se) into proteins.^1^^,^^2^ Selenoproteins contain selenocysteine,^3^ a rare amino acid incorporated during translation in response to an in-frame UGA codon, otherwise considered a termination codon.^4^ Recoding involves a selenocysteine insertion sequence (SECIS) element in the mRNAs 3′ UTR, forming a hairpin structure. This process requires SECIS binding protein 2 (SECISBP2) and the selenocysteine tRNA-specific eukaryotic elongation factor (EEFSEC), which together bind Sec-tRNA^Sec^ to the UGA codon in the ribosomal A-site. The biosynthesis of Sec on its cognate tRNA^Sec^ is conserved across bacteria, archaea, and eukaryotes (Figure 1).^3^^,^^5^^,^^6^^,^^7^^,^^8^^,^^9^^,^^10^^,^^11^ The human genome encodes 25 known selenoproteins, with EEFSEC playing a pivotal role in the final step of Sec incorporation.^10^^,^^12^^,^^13^ EEFSEC, in complex with Sec-tRNA^Sec^, delivers Sec to the ribosome during translation. Both EEFSEC and tRNA^Sec^ are essential for selenoprotein biosynthesis in the human body (Figure 1).^7^^,^^8^^,^^14^^,^^15^

Selenoproteins perform various biological functions, including the metabolism of thyroid hormones through Sec-containing deiodinases^4^ and antioxidant activities via glutathione peroxidases (GPXs), thioredoxin reductases (TXNRDs), and methionine sulfoxide reductase B1 (MSRB1).^16^^,^^17^^,^^18^^,^^19^^,^^20^^,^^21^ Selenoproteins are also involved in selenium transport and delivery (selenoprotein P [SELENOP]), protein folding and endoplasmic reticulum (ER) stress (SELENOF, SELENOM, SELENOS), phospholipid biosynthesis (SELENOI),^22^^,^^23^ and Ca^2+^ handling in the ER (SELENON, SELENOT).^24^

Neurodegenerative multisystem disorders related to selenoproteins and their biosynthesis include autosomal recessive pontocerebellar hypoplasia type 2D due to SEPSECS deficiency^13^^,^^25^ (*SEPSECS* [MIM: 613811]), Sedaghatian-type spondylometaphyseal dysplasia due to GPX4 deficiency (*GPX4* [MIM: 250220]),^26^ TXNRD1 deficiency (*TXNRD1* [MIM: 601112]),^27^ and autosomal recessive spastic paraplegia 81 due to selenoprotein I deficiency (*SELENOI* [MIM: 618768]).^22^^,^^23^ Selenoprotein-related disorders not involving the nervous system are characterized by impaired thyroid hormone metabolism, such as in deficiency of SECISBP2 (*SECISBP2* [MIM: 609698])^28^^,^^29^ and deficiency of SELENON (*SELENON* [MIM: 602771]),^30^ where affected individuals show primarily compromised muscle function. Isolated deficiency of TXNRD2 leads to glucocorticoid deficiency (*TXNRD2* [MIM: 617825]) without any reported neurological, thyroid, or muscular symptoms.^31^

This study describes nine individuals from eight unrelated families with an early-onset neurodegenerative disorder due to bi-allelic *EEFSEC* variants (MIM: 607695), thereby expanding the spectrum of inborn errors of selenoprotein metabolism.

Clinical procedures adhered to Helsinki Declaration principles, and informed consent of parents and guardians was obtained (supplemental information). Exome or genome sequencing was performed on individuals with early-onset neurodegenerative disorders as described before (supplemental information).^32^^,^^33^^,^^34^^,^^35^^,^^36^ The cohort was assembled via GeneMatcher and personal communication.^37^

**Figure 2:**
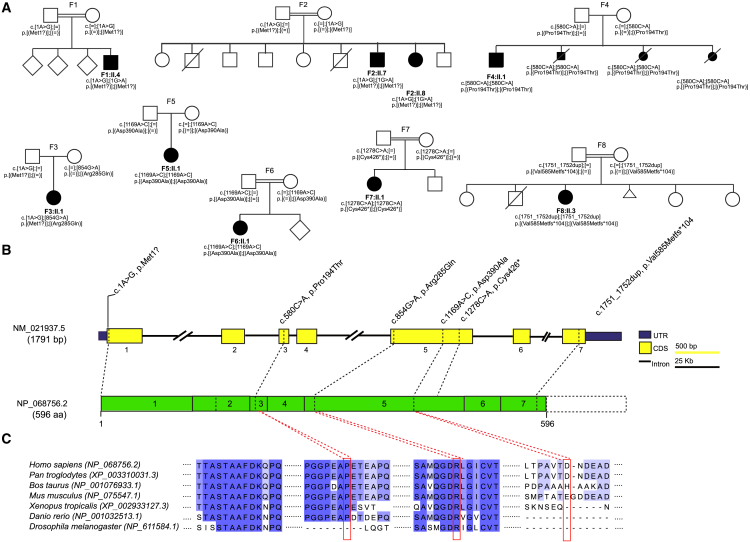
Pedigrees of investigated families and structure of EEFSEC (A) Pedigrees of eight families with pathogenic variants in *EEFSEC*, illustrating the variant carrier status of affected (closed symbol) and healthy (open symbol) family members. Unaffected siblings were not tested unless indicated. F1:II.4, F2:II.7, and F2:II.8 harbor a homozygous start-loss variant (c.1A>G [p.Met1?]), and F3:II.1 was found to be compound heterozygous for the same start-loss variant (c.1A>G [p.Met1?]) and another missense variant in exon 5 (c.854G>A [p.Arg285Gln]). In family F4 (F4:II.1 and 3 affected fetuses), F5:II.1 and F6:II.1 missense variants were detected in homozygous states, respectively, in exon 3 (F4:II.1: c.580C>A [p.Pro194Thr]) and exon 5 (F5:II.1 and F6:II.1: c.1169A>C [p.Asp390Ala]). F7:II.1 harbors a homozygous nonsense variant (c.1278C>A [p.Cys426^∗^]) leading to either nonsense-mediated mRNA decay or a loss of 170 amino acids. In family F8 (F7:II.3), a homozygous frameshift variant was identified (c.1751_1752dup [p.Val585Metfs^∗^104]), expected to lead to a protein extension of 120 amino acids. All variants were either absent or listed exclusively in a heterozygous state in gnomAD 4.0 at the time of reporting. (B) Structure of *EEFSEC* and the encoded protein with known domains and position of identified variants. CDS, coding sequence; UTR, untranslated region. (C) Conservation of variants in *EEFSEC* across vertebrate and invertebrate animals.

Bi-allelic variants in *EEFSEC* (GenBank: NM_021937.5) were detected in nine individuals with an early-onset neurodegenerative disorder from six different countries and ethnic groups (Table 1). Sanger sequencing confirmed full co-segregation of highly conserved *EEFSEC* variants with the clinical phenotype across all families (Figures 2A and 2B).

**Table 1:** Clinical disease features in EEFSEC deficiency

**Individual**	**F1:II.4**	**F2:II.7**	**F2:II.8**	**F3:II.1**	**F4:II.1**	**F5:II.1**	**F6:II.1**	**F7:II.1**	**F8:II.3**
Gender	male	male	female	female	male	female	male	female	female
Country of origin	Brazil	Brazil	Brazil	US	Georgian Jewish	Turkey	Turkey	Pakistan	Afghanistan
cDNA change(s)	c.1A>G	c.1A>G	c.1A>G	c.1A>G, c.854G>A	c.580C>A	c.1169A>C	c.1169A>C	c.1278C>A	c.1751_1752dup
Protein change(s)	p.Met1?	p.Met1?	p.Met1?	p.Met1?, p.Arg285Gln	p.Pro194Thr	p.Asp390Ala	p.Asp390Ala	p.Cys426^∗^	p.Val585Metfs^∗^104
Allelic status	homozygous	homozygous	homozygous	compound heterozygous	homozygous	homozygous	homozygous	homozygous	homozygous
Age at onset/last examination	congenital/20 yrs	congenital/yrs	congenital/21 yrs	congenital/8 yrs	congenital/6 yrs	congenital/3. yrs	congenital/17 mths	congenital/11 yrs	congenital/16 yrs
First clinical features	club feet, developmental delay	developmental delay	developmental delay	microcephaly, developmental delay	severe muscular hypotonia, respiratory distress	IUGR, developmental delay	IUGR, developmental delay	dysphagia	severe muscular hypotonia
Microcephaly	secondary	primary	primary	primary	secondary	primary, progressive	secondary	secondary	secondary
Cognitive impairment	moderate	severe	severe	moderate	severe	severe	moderate	severe	severe
Delay of speech development	+	+	+	+	no expressive language	+	+	+	no expressive language
Delay of motor development (age walking)	+	+ (not achieved)	+ (not achieved)	+ (14 mths)	+ (not achieved)	+ (22 mths)	+	+ (35 mths)	+ (not achieved)
Spasticity	progressive, predominantly lower extremities	progressive, predominantly lower extremities	progressive predominantly lower extremities	progressive, predominantly lower extremities	progressive	–	–	progressive predominantly lower extremities	progressive
Ataxia	dysmetric jerks, nystagmus	nystagmus, saccadic breakdown	–	intention tremor, dysarthria, atactic gait	–	–	–	atactic gait, intention tremor	–
Peripheral neuropathy	N/D	N/D	+	N/D	N/D	N/D	N/D	+	N/D
Seizure semiology (age at onset)	tonic-clonic (19 yrs)	complex focal (10 yrs)	complex focal (2 yrs)	complex focal, repetitive status epilepticus (18 mths)	+	–	–	myoclonic seizures (3–4 yrs)	complex focal (2 ds)
Vision impairment/hearing impairment	−/−	strabism/−	strabism/−	−/−	both N/D	oculomotor apraxia, horizontal gaze palsy/+	apraxia, horizontal gaze palsy/+	−/−	optic nerve hypoplasia/−
Other	club feet	micrognathia, facial dysmorphism, hypochromic skin lesion	thin upper lip, hypochromic skin lesion	bilateral mild 5th finger clinodactyly, slight 2–3 syndactyly of toes	severe contractures, club feet	syndactyly, nail dysplasia	joint laxity, facial dysmorphism, high arched palate	–	facial dysmorphism, microphthalmia, dysmorphic ears, nail dysplasia

+, present on examination; −, absent on examination; N/D, not done; wks, weeks; yrs, years; mths, months; ds, days; IUGR, intrauterine growth restriction.

Affected individuals showed primary global developmental delay and microcephaly. Most of the children developed progressive spasticity, mainly in the lower extremities, and complex epilepsy with variable onset in childhood. Several developed an atactic movement disorder during the course of the disease. Cognitive development varied within the cohort, ranging from primary stagnation to moderate delay (Table 1). Peripheral neuropathy was present in those examined. Two individuals had visual impairment, with one diagnosed with bilateral optic nerve hypoplasia (F8:II.3) (Table 1; supplemental information). Among the nine affected individuals, a moderate and a severe phenotype could be distinguished: two infants showed a severe phenotype (F4:II.1, F8:II.3) with severe truncal hypotonia, respiratory distress, or postnatal seizures, resulting in minimal developmental progress without attainment of basic skills. Seven individuals exhibited a moderate phenotype, achieving some motor and cognitive milestones with variable delays (Table 1; supplemental information). Six affected individuals had normal pregnancy and birth histories; two had intrauterine growth restriction and were born preterm (F5:II.1 and F6:II.1). In family F4, three pregnancies were terminated due to severe contractures and cerebellar hypoplasia detected by prenatal ultrasound (Figure S1). Five individuals presented with congenital malformations such as clubfeet, contractures, or syndactyly. Laboratory investigations, where available, showed normal selenium levels and thyroid function (supplemental information; Table S1).

**Figure 3:**
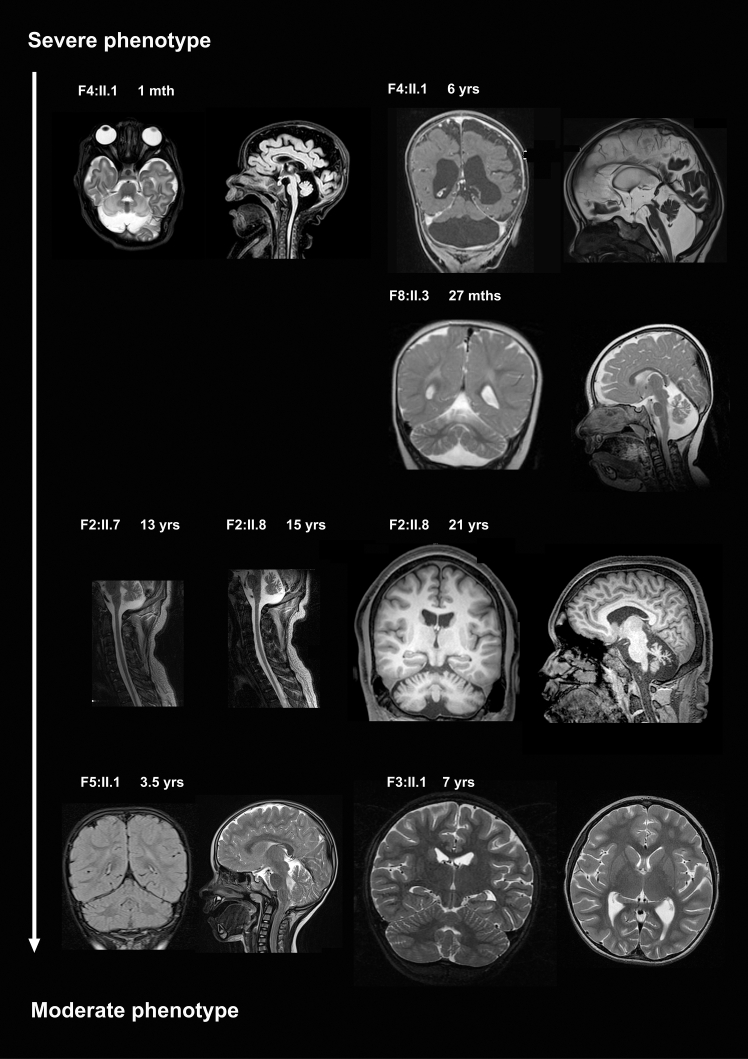
Neuroimaging features of EEFSEC deficiency Representative MR images of the brain show distinct pathological features in individuals with EEFSEC deficiency, distinguishing between severe and moderate phenotypes. The severe phenotype includes cerebellar hypoplasia (F4:II.1, top row) and marked cerebellar atrophy (F8:II.3, second row, and F4:II.1, top row). At 1 month of age, the cerebellar hemispheres and vermis are small, and the cervical spinal cord is slender (far left T2w axial and left FLAIR sagittal); at 6 years of age, the coronal T1w image (top right row) shows cerebellar hypoplasia with disturbed cerebellar proportion (dragonfly appearance) and malformed, short cerebellar folia with poorly identifiable branching, and the cerebrum appears dysplastic and atrophic; the sagittal T2w image (far right) shows the small vermis unchanged. Myelination is severely delayed. F8:II.3 (second row, far left T2w coronal and left sagittal) at 27 months shows more severe cerebellar atrophy affecting the vermis but also the hemispheres: cerebellar volume is reduced but its proportions remain normal. The rarefaction of the folia leads to enlarged sulci, with a skeletal appearance of the vermis. F2:II.7 (third row, far left sagittal T2w image) at 13 years shows cerebellar atrophy illustrated in the vermis and a thin cervical spinal cord; F2:II.8 (left sagittal T2w image and right and far right sagittal and coronal T1w images, respectively) at 15 years shows similar vermis atrophy, and at 21 years, the atrophy has reached a more skeletal appearance (so the atrophy appears to be progressive), while the hemispheres show less pronounced atrophy. F5:II.1 (bottom row, far left sagittal T2w image) at 3.5 years shows mild vermis atrophy, while the cerebrum appears normal, whereas in F3:II.1 (right and far right coronal and axial T2w images, respectively), at 7 years, the cerebellum appears normal, and only the posterior ventricles are slightly enlarged, indicating some cerebral atrophy. mths, months; yrs, years.

MR images were obtained from ages 1 month to 21 years. Seven brain MRI and two spinal cord MRI datasets were re-analyzed, independently assessed by at least two neuropediatricians or neuroradiologists (supplemental information; Table S2). Neuroimaging assessments across ages revealed phenotypic profiles linked to the clinical spectrum (Figure 3). The severe phenotype included disrupted brain development, cerebellar hypoplasia, and dysplastic cerebrum with delayed myelination (F4:II.1), along with pronounced cerebellar atrophy and delayed myelination (F8:II.3). The moderate phenotype showed mild to moderate cerebellar atrophy. Consistent patterns included a preference for cerebellar involvement, particularly affecting the vermis more than the hemispheres. Some had a thin cervical spinal cord, unrelated to cerebral severity. Few individuals displayed mild cerebral atrophy, sparing the cerebellum.

**Figure 4:**
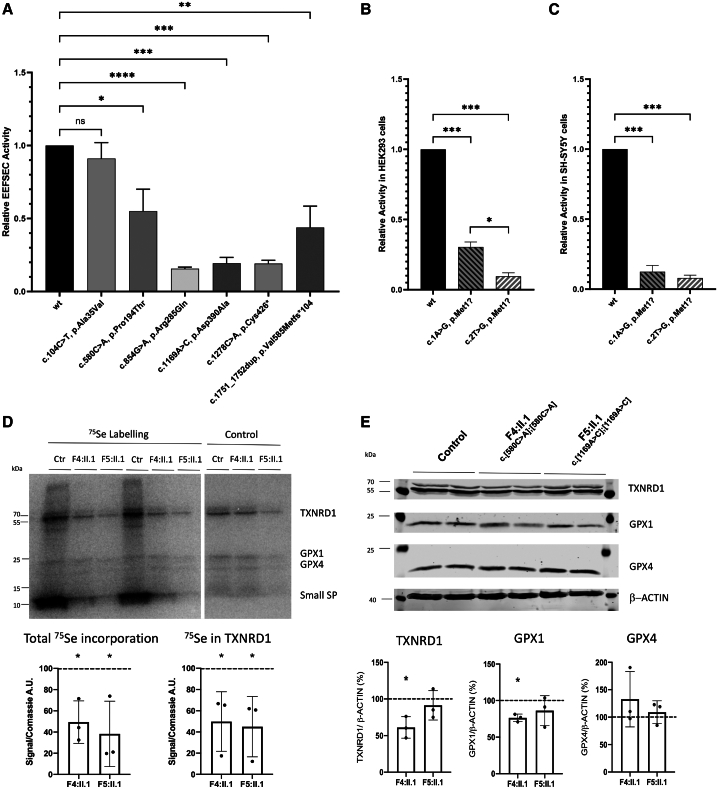
*In vitro* studies on the fuction of EEFSEC (A–C) *In vitro* analysis of the *EEFSEC* variants in comparison to wild-type EEFSEC. (A) The relative luciferase (luc) activity of a UGA/Sec-containing luc translated in a cell-free system and dependent on addition of recombinant *EEFSEC* variants was significantly reduced compared to the wild type for all identified *EEFSEC* variants (c.580C>A [p.Pro194Thr]; c.854G>A [p.Arg285Gln]; c.1169G>A [p.Asp390Ala]; c.1278C>A [p.Cys426^∗^]; and c.1751_1752dup [p.Val585Metfs^∗^104]) except for one likely benign variant, c.104C>T (p.Ala35Val) (supplemental information). (B and C) Relative luc activity of an EEFSEC-luc in HEK293 cells (B) or SH-SY5Y cells (C) carrying either wild-type or mutated initiation codons. One-way ANOVA, followed by Dunnett's multiple comparisons test. *n* = 3 experiments; n.s., not significant; ^∗^*p* < 0.05, ^∗∗^*p* < 0.01, ^∗∗∗^*p* < 0.001. Reduced EEFSEC-luc translation is observed for both start-loss variants, but the c.1A>G (p.Met1?) variant identified in families F1, F2, and F3 shows cryptic initiation of translation. The data of (A)–(C) are represented as mean ± SEM. Representative ^75^Se incorporation of selenoprotein (SP) expression in EFFSEC-deficient fibroblast cell lines. (D) Human fibroblasts were metabolically labeled with ^75^SeO32-. Major ^75^Se-labeled bands are indicated and identified as TXNRD1, GPX1, GPX4, and small SPs. Patient-derived fibroblasts showed a significant reduction of incorporation of ^75^Se compared to control cell lines in total protein and TXNRD1. *n* = 3 experiments. (E) Western blots against representative SPs in human fibroblasts. *n* = 3 experiments. Graphs show the percentage of protein expression relative to healthy control cells (set at 100%). The signals are normalized to b-ACTIN expression.

*In silico* modeling showed no major structural changes or destabilization for all *EEFSEC* variants. However, the c.854G>A (p.Arg285Gln) variant is predicted to show reduced binding to Sec (Figure S2; supplemental information). The impact of the identified variants on protein function was assessed using an *in vitro* activity assay for EEFSEC (supplemental information).^7^ Activity of the UGA/Sec-containing luciferase (luc) reporter construct was significantly reduced for all *EEFSEC* variants compared to the wild type, with the exception of the likely benign variant c.104C>T (p.Ala35Val) identified in additional family 9 (Figure 4A). To investigate potential cryptic translational activity of the start-loss variant (c.1A>G [p.Met1?]), an EEFSEC-luc fusion construct was expressed in cultured cells, which contained the start-loss variant (GUG, identified in F1, F2, and F3) or an AGG codon (c.2T>G) instead of the start codon (supplemental information). While the start-loss variant (c.1A>G [p.Met1?]) maintained some cryptic initiation in HEK293 cells greater than the AGG variant (Figure 4B), in neuronal SH-SY5Y cells, both initiation codon mutants presented negligible activity.

Metabolic labeling with ^75^Se of patient-derived fibroblasts was used to demonstrate a global impairment of selenoprotein biosynthesis in EEFSEC deficiency. Total ^75^Se incorporation was reduced by more than half compared to control in two patient cell lines (F4:II.1 and F5:II.1; supplemental information).^38^ A similar reduction of ^75^Se incorporation was also found in the highly expressed selenoprotein TXNRD1, which carries Sec as penultimate amino acid (Figure 4D). Western blotting was used to quantify selenoproteins GPX1, GPX4, and TXNRD1 but was less sensitive and revealed significantly reduced GPX1 and TXNRD1 only in one EEFSEC-deficient cell line (Figure 4E). GPX4 signals were not significantly reduced, but the growth of EEFSEC-deficient fibroblast cell lines was enhanced in the presence of the ferroptosis inhibitor liproxstatin (Figure S3; supplemental information).

**Figure 5:**
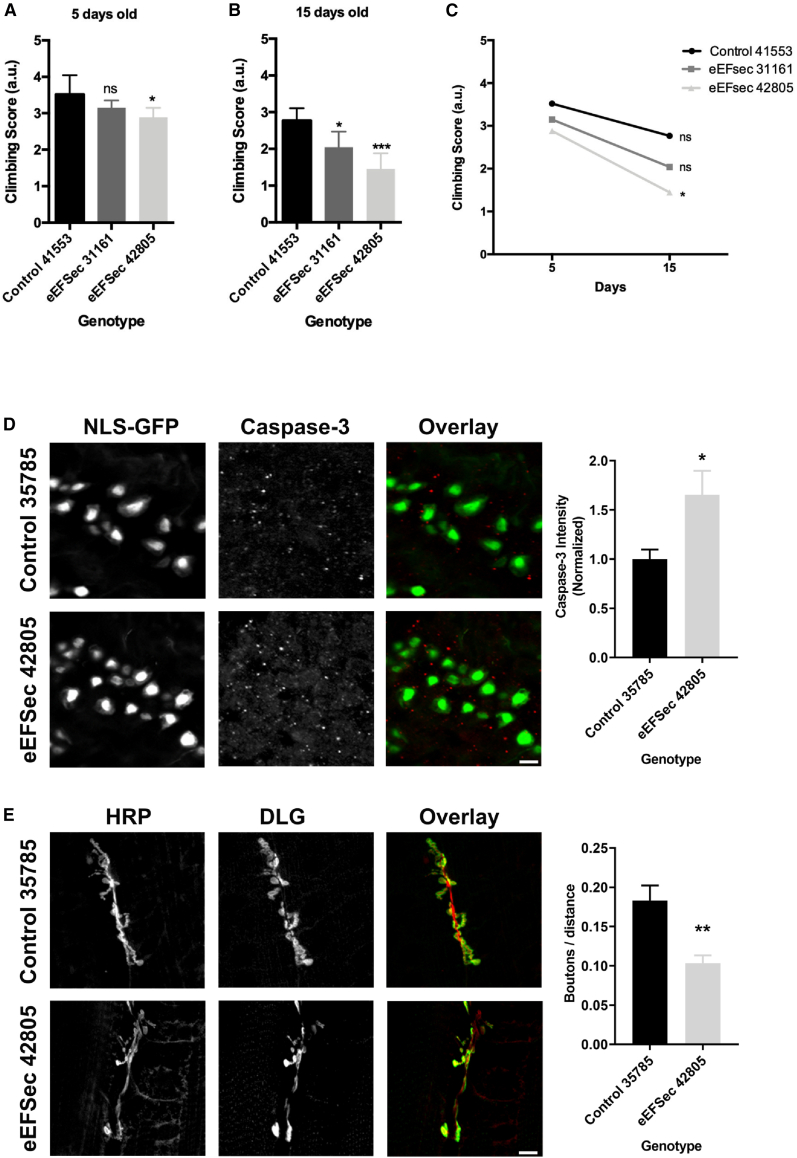
Functional analyses of eEFSec in *Drosophila* (A–C) Quantification of climbing performance of flies expressing eEFSec-RNAi in motor neurons. Climbing score analysis at 5 s. (A and B) Climbing score means of 5 (A) and 15 days of age (B). (C) Graph shows the mean differences between day 5 and 15 for each genotype. (two-way ANOVA, Bonferroni’s multiple comparisons test). Note that flies expressing eEFSec-RNAi 42805 display a progressive reduction of climbing performance. Repeated-measure (RM) one-way ANOVA, with the Geisser-Greenhouse correction, followed by Dunnett's multiple comparisons test. n.s., not significant; ^∗^*p* < 0.05, ^∗∗^*p* < 0.01, ^∗∗∗^*p* < 0.001, and ^∗∗∗∗^*p* < 0.0001. (D) The level of cleaved caspase-3 was evaluated by immunofluorescence using an anti-cleaved caspase-3 antibody in *Drosophila* larval brains expressing eEFSec RNAi (42805) or its control (35785), as well as an NLS-GFP (OK6-Gal4>NLS-GFP) to visualize motor neurons. Graph shows the normalized fluorescence values between genotypes (*N* = 14 brains analyzed in control animals and 12 brains for eEFSec RNAi-expressing animals). Mann-Whitney test, ^∗^*p* < 0.05. Scale bar: 10 μm. (E) Changes in synaptic boutons of motor neurons were assessed by counting the number of DLG-positive postsynaptic terminals in *Drosophila* larvae body wall muscle preparations expressing eEFSec RNAi (42805) in motor neurons or its control (35785). In addition, samples were stained with anti-HRP antibody to visualize the motor neuron membrane. Graph show the number of boutons (divided by the distance the analyzed motor neuron membrane) between genotypes (*N* = 14–18 from 8 animals per genotype). Mann-Whitney test, ^∗∗^*p* < 0.01. Scale bar: 7 μm.

To assess the functional consequences of EEFSEC deficiency, an eEFSec-RNAi *Drosophila* model was employed (supplemental information).^39^ Two different RNAis targeting eEFSec were expressed specifically in motor neurons using specific C380-Gal4 drivers to test whether decreased eEFSec function affects locomotion and cellular phenotypes. Climbing performance was evaluated in 5- and 15-day-old flies to examine potential progressive motor defects. At 5 days old, only RNAi line eEFSec 42805 showed decreased climbing performance. At 15 days old, both RNAi lines affected climbing. The effect of RNAi line eEFSec 42805 was more pronounced at 15 days, suggesting a progressive decline in motor function with reduced eEFSec activity in motor neurons (Figures 5A–5C). Accordingly, expression of both eEFSec RNAis in larval motor neurons result in increased caspase-3 staining, an effector of the apoptotic program (Figure 5D; supplemental information).^40^ Moreover, eEFSec knockdown using RNAi line eEFSec 42805 results in a decreased number of synaptic boutons in the neuromuscular junction (Figure 5E). These data together suggest that eEFSec knockdown promotes cell death and synaptic defects affecting neuronal function.

In conclusion, bi-allelic *EEFSEC* variants that impacted selenoprotein expression were identified in eight unrelated families, leading to selenoprotein deficiency and neurodegenerative disease. All affected individuals showed moderate to profound intellectual disability and microcephaly. Most developed progressive spasticity and complex seizures during childhood. Neuroimaging data revealed mainly cerebellar pathology of varying severity, affecting the vermis more than the hemispheres. Cerebellar atrophy was seen in several affected individuals, with progression shown in one individual at longer follow-up. In the most severely affected child, cerebellar hypoplasia indicated prenatal onset of neurodegeneration, with severely delayed myelination highlighting the severity of disturbed neurodevelopment.

In a cell-free translation system, all pathogenic *EEFSEC* variants exhibited significantly reduced UGA/Sec recoding in the luc reporter compared to the wild-type control, indicating impaired selenoprotein translation. The truncating variant c.1278C>A, p.Cys426^∗^ identified in F7:II.1 seems more severe than the C-terminal extension observed in F8:II.3, which retains some intermediate EEFSEC activity. Using an EEFSEC-luc reporter system, cryptic initiation at the mutated initiation codon identified was demonstrated, suggesting low expression of EEFSEC protein from the c.1A>G allele. Although the start-loss variant may present a milder phenotype compared to other variants, a definitive genotype-phenotype correlation cannot be established based on this small cohort.

An open question is why the *EEFSEC* deficiency primarily affects the brain. Selenoprotein expression in fibroblasts is only moderately altered, consistent with the absence of pathology outside the brain. Interestingly, the experiments on cryptic translation initiation suggested that HEK293 cells were more efficient in initiation EEFSEC than neuronal SH-SY5Y cells. Similarly, a mouse model for Sepsecs deficiency demonstrated massive effects on selenoprotein expression in the brain and in cultured neurons but not in the liver or kidney.^38^ As in this mouse model, plasma selenium (or SELENOP), which is secreted by the liver, is not reduced in affected individuals.

The knockdown of eEFSec displays similar phenotypes to hereditary spastic paraplegia models in *Drosophila*, which show decreased motor function and synaptic defects at larval and adult stages.^41^^,^^42^^,^^43^^,^^44^ However, the role of eEFSec has not been studied in this context. *Drosophila* eEFSec has around 50% identity with mammalian orthologs and is expressed from embryo to adult stages.^45^^,^^46^ An eEFSec null mutation generated by deleting the N-terminal region does not enhance oxidative stress effects on lifespan.^46^ Our result showing a decrease in the number of synaptic boutons in the larval neuromuscular junction, together with the diminished dendritic branching observed in flies expressing RNAis targeting protein initiation and elongation factors, including eEFSec, in *Drosophila* sensory neurons, support an eEFSec role in neuron differentiation.^47^ This evidence, together with increased caspase-3 staining in larval motor neurons in flies expressing eEFSec RNAis, supports the hypothesis that eEFSec’s function in neuron differentiation contributes to progressive effects in the nervous system. However, further research is needed to confirm whether the progression of motor dysfunction is initiated during development or neuron maintenance in the *Drosophila* model.

Human selenoprotein deficiencies can lead to endocrine disorders, cardiovascular issues, altered immune response, myopathy, or severe neurodegenerative phenotypes.^48^^,^^49^^,^^50^^,^^51^ Protection from reactive oxygen species (ROS) relies on different GPXs and TXNRDs that are relevant for the recycling of antioxidants, including vitamin C and E as well as coenzyme Q.^48^^,^^49^^,^^50^ Impaired selenoprotein-dependent ROS clearance might be particularly relevant in the brain, with metabolically highly active cells. Along this line, several fibroblast cell lines failed to grow despite multiple skin biopsy trials and selenium-enriched culture media. One fibroblast cell line grew very slowly until the ferroptosis inhibitor liproxstatin was added to the culture media, suggesting that GPX4 deficiency causes the survival defect (Figure S3).^52^^,^^53^^,^^54^

The clinical phenotype and neuroimaging of EEFSEC-deficient probands resembles findings from affected individuals with SEPSECS deficiency. Early-onset SEPSEC deficiency is notably associated with cerebellar hypoplasia.^13^^,^^55^ Affected individuals exhibit mild to profound intellectual disability, progressive microcephaly, and spasticity. Seizures often develop during infancy. Despite initial reports of severe neurodegeneration, recent studies show a broad clinical spectrum, including late-onset, slowly progressing cerebellar atrophy.^25^^,^^56^ Lactacidemia due to mitochondrial dysfunction, seen in SEPSECS deficiency, was observed only infrequently in EEFSEC deficiency.^55^ Intractable seizures, as seen in one case, have been reported in Se-responsive epilepsy and TXNRD1 deficiency.^27^^,^^57^ Seizures, spasticity, and atactic gait were primary phenotypes in *Selenop*-deficient mice^58^ and SELENOP-deficient dogs.^59^ In these animal models, selenium levels in the brain are severely reduced. Less is known about how selenoprotein I deficiency causes a similar but variable phenotype, with progressive spasticity as a common feature.^22^^,^^23^

Another selenopathy is caused by autosomal recessive SECISBP2 deficiency presenting with a complex clinical spectrum with abnormal thyroid function tests (elevated TSH and free T4, low free T3) and myopathy^29^ in some individuals with developmental delay.^60^ Myopathy has not been observed clinically in EEFSEC deficiency, although affected fetuses showed non-specific muscle changes pathologically (supplemental information). In SECISBP2 deficiency, secondary effects from ROS-mediated damage can include increased photosensitivity, azoospermia, defective T cell maturation, and aortic aneurism.^29^^,^^51^ These findings were not reported in EEFSEC deficiency. The neurological phenotype of SECISBP2 deficiency varies, from isolated myopathy to severe developmental delay and progressive sensorineural hearing loss (SNHL).^60^^,^^61^^,^^62^ SNHL has not yet been reported in EEFSEC deficiency, possibly due to the lack of a long-term follow-up, as most affected individuals are still children. In contrast to SECISBP2 deficiency, thyroid hormone levels, surrogate markers for altered Sec-containing deiodinase activity, and selenium levels, indicating disturbed plasma GPXs, were within the normal range in the EEFSEC-deficient probands in this study. However, comprehensive biomarker investigations will be crucial in future studies involving larger cohorts to identify subtle biochemical alterations.

Bi-allelic variants in tRNA^Sec^ (*TRU-TCA1-1* [MIM: 165060]) cause a syndrome similar to SECISBP2 deficiency.^63^ Affected individuals may exhibit myopathy, thyroid hormone dysfunction, and increased ROS levels in fibroblasts. Proteome and transcriptome analyses decipher changes in stress-induced selenoproteins but not in housekeeping selenoproteins.^63^ The abnormal thyroid function tests in *SECISBP2*- and *TRU-TCA1-1*-deficiency are caused by impaired expression of deiodinases. The Sec-containing iodothyronine deiodinases DIO1 and DIO2 convert thyroxine (T4) to its active form, triiodothyronine (T3), while DIO3 inactivates both T3 and T4 to T2 and rT3, respectively. Where thyroid function tests were available, we did not find changes in EEFSEC-deficiency—a similar yet unexplained observation as in SEPSECS deficiency. If the pathology caused by impaired selenoprotein biosynthesis is, at least in part, caused by the inefficient removal of ROS, and potentially by cell demise through ferroptosis, then any treatment with (lipophilic) antioxidants or inhibitors of ferroptosis may be worth future exploration in order to treat the condition.

## Data and code availability

This study did not generate new code. Sequence datasets have been generated and contributed by different study sites and have not been deposited in a public repository due to varying local consent regulations. Selected datasets might be available from the corresponding author on request.

## Acknowledgments

First and foremost, the authors express their gratitude to the families who participated in the study. Additionally, acknowledgments are given to Miljan Simonovic for kindly providing the EEFSEC plasmid and Paul Copeland for the luc^64^ construct. We appreciate the contributions of Philip Micheel, Maura R.Z. Ruzhnikov, and Lucia Ines Macedo-Souza to clinical management. Additionally, we acknowledge Bernt Schulze, Adriana Rebelo, Hilal Yıldız Er, and Fernando Kok for providing genetic data. U.S. is supported by Universitätsklinikum Bonn. T.B.H. is supported by the 10.13039/501100001659Deutsche Forschungsgemeinschaft (DFG; German Research Foundation – 418081722 and 433158657), and the 10.13039/501100000780European Commission (Recon4IMD - GAP-101080997). R.B. is supported by Deutsche Forschungsgemeinschaft (German Research Foundation, DFG, grant number BU 3602/1-1). D.S.L. is supported by the National Institute for Health and Care Research University College London Hospital’s Biomedical Research Centre. M.U. is supported by the 10.13039/501100013302King Abdullah International Medical Research Center (KAIMRC), Riyadh, 10.13039/100019223Kingdom of Saudi Arabia (KSA) (NRC23R/177/02). P.J.T. was supported by an 10.13039/501100000265MRC strategic award to establish an International Centre for Genomic Medicine in Neuromuscular Diseases (ICGNMD) MR/S005021/1.

## Author contributions

L.L., R.B., and T.B.H. designed the experiments. R.B., M.A.E., A.J.M., O.R., W.M., P.J.T., G.A.R., M.M., S.M., M.B., S.Z., D.L., O.R., Z.G.-O., and T.B.H. conducted genetic studies and contributed to the interpretation of clinical data. M.A. and B.M. performed *in silico* modeling. M.S. performed the bioinformatic analysis. L.L., S.M., S.S., M. Fuchs, D.L., D.S.L., U.D., M.F., F.F., U.S.M., L.M., O.S., S.A., K.Y., Z.L., H.R., M.U., E.U., A.Y., O.O., T.C., and D.L. carried out phenotyping or collected clinical data. L.L., B.B., S.G., and I.K.-M. re-analyzed the MRI data. R.B., J.E., S.P.M., A.G., C.S., C.K., L.S., U.S., and M. Fabiano performed and analyzed cell culture and bacteria experiments. P.O., J.A., N.C., and A.G.-G. conducted and analyzed *Drosophila* experiments. L.L., R.B., and T.B.H. wrote the initial version of the manuscript. All authors contributed to reviewing and editing the manuscript.

## Declaration of interests

B.B. is a co-founder, shareholder, and CTO of AIRAmed GmbH.

## Web resources

Allen Brain Map, https://portal.brain-map.org

Combined Annotation Dependent Depletion 23, https://cadd.gs.washington.edu

Ensembl Variant Effect Predictor, https://ensembl.org/Homo_sapiens/Tools/VEP

gnomAD server, https://gnomad.broadinstitute.org

GTEx Portal, https://gtexportal.org

The Human Brain transcriptome, https://hbatlas.org/pages/hbtd

The Human Protein Atlas, https://www.proteinatlas.org

NMDEscPredictor, https://nmdprediction.shinyapps.io/nmdescpredictor/

Online Mendelian Inheritance in Man (OMIM), https://www.omim.org

UCSC (GRCh38/hg38 assembly), https://genome.ucsc.edu

## Supplemental information

Document S1. Figures S1–S5, Tables S1 and S2, supplemental notes, and supplemental methods

Document S2. Article plus supplemental information
